# Metabolic surgery-induced changes of the growth hormone system relate to improved adipose tissue function

**DOI:** 10.1038/s41366-023-01292-7

**Published:** 2023-03-23

**Authors:** Sofiya Gancheva, Sabine Kahl, Christian Herder, Klaus Strassburger, Theresia Sarabhai, Kalliopi Pafili, Julia Szendroedi, Matthias Schlensak, Michael Roden

**Affiliations:** 1 Department of Endocrinology and Diabetology, Medical Faculty and University Hospital, Heinrich-Heine University, Düsseldorf, Germany; 2 Institute for Clinical Diabetology, German Diabetes Center, Leibniz Center for Diabetes Research at Heinrich Heine University, Düsseldorf, Germany; 3 German Center for Diabetes Research (DZD e.V.), Partner Düsseldorf, Munich-Neuherberg, Düsseldorf, Germany; 4 Institute for Biometrics and Epidemiology, German Diabetes Center, Leibniz Center for Diabetes Research at Heinrich Heine University, Düsseldorf, Germany; 5 Department of Internal Medicine I and Clinical Chemistry, Heidelberg University Hospital, Heidelberg, Germany; 6 Obesity and Reflux Center, Neuwerk Hospital, Mönchengladbach, Germany

**Keywords:** Obesity, Obesity

## Abstract

**Aims:**

Body weight loss improves insulin resistance and growth hormone secretion in obesity, which may be regulated by leptin according to preclinical studies. How changes in leptin, lipids and insulin sensitivity after bariatric (metabolic) surgery affect the human growth hormone system is yet unclear.

**Participants and methods:**

People with obesity (OBE, *n* = 79, BMI 50.8 ± 6.3 kg/m^2^) were studied before, 2, 12, 24 and 52 weeks after metabolic surgery and compared to lean healthy humans (control; CON, *n* = 24, BMI 24.3 ± 3.1 kg/m^2^). Tissue-specific insulin sensitivity was assessed by hyperinsulinemic-euglycemic clamps with D-[6,6-^2^H_2_]glucose. Fasting leptin, growth hormone (GH), insulin-like growth factor 1 (IGF-1) and IGF-binding proteins (IGFBP1, IGFBP3) were measured using ELISA.

**Results:**

At baseline, OBE exhibited higher glycemia and leptinemia as well as pronounced peripheral, adipose tissue and hepatic insulin resistance compared to CON. GH and IGFBP1 were lower, while IGF1 was comparable between groups. At 52 weeks, OBE had lost 33% body weight and doubled their peripheral insulin sensitivity, which was paralleled by continuous increases in GH, IGF-1 and IGFBP1 as well as decrease in leptin. The rise in GH correlated with reductions in free fatty acids, adipose tissue insulin resistance and insulinemia, but not with changes in body weight, peripheral insulin sensitivity, glycemia or leptinemia. The rise in IGF-1 correlated with reduction in high-sensitive C-reactive protein.

**Conclusion:**

Reversal of alterations of the GH-IGF-1 axis after surgically-induced weight loss is unlikely related to improved leptin secretion and/or insulin sensitivity, but is rather associated with restored adipose tissue function and reduced low-grade inflammation.

## Introduction

The rising prevalence of obesity and its associated complications such as type 2 diabetes (T2D), cardiovascular disease or cancers is becoming an increasing burden to healthcare systems globally [1]. In addition, endocrine disorders such as thyroid dysfunction [2] and particularly impaired growth hormone (GH) secretion [3] have been linked to the obesity epidemic.

Body weight loss improves insulin sensitivity even leading to remission of T2D, but may also normalise GH secretion by yet unclear mechanisms [4]. Effective weight loss upon bariatric (metabolic) surgery has been shown to profoundly alter gastrointestinal hormones controlling glucose and energy homoeostasis [5] but also to increase circulating GH concentrations. In contrast, cross-sectional studies on its impact on circulating insulin-like growth factor 1 (IGF-1) revealed conflicting results, by showing unchanged [6–8], decreased [9, 10] or even increased concentrations [11, 12] in lean humans compared with people with obesity. IGF-1 is involved in the regulation of both GH and insulin secretion to promote physiological carbohydrate and lipid metabolism [13], but its contribution to the improvement in tissue-specific insulin sensitivity after bariatric surgery also remains unclear.

The complex regulation of the GH-IGF-1 axis includes hypothalamic neuropeptides, ghrelin, insulin, free fatty acids (FFA), nutritional factors and IGF1-binding proteins (IGFBPs) [14]. Leptin, a key signal of long-term energy availability and an indicator of fat mass, inhibits GH secretion [15] and has been implicated in the regulation of IGF-1 secretion [16]. Indeed, recent studies showed that leptin substitution in children increases IGF-1 levels [17]. Furthermore, improvement of insulin sensitivity after metabolic surgery determines the restoration of leptin sensitivity through a molecular mechanism involving fatty acid-control of muscle malonyl-Co-A synthesis [15], indicating a direct link between leptin levels and lipid availability. Furthermore, increased muscle lipid oxidation pathways and regulation of muscle differentiation in people with obesity at 52 weeks after metabolic surgery [18] could relate to improved leptin and GH secretion. However, a direct association and possible mediators have not been demonstrated so far.

Previous studies were performed on rather small cohorts without detailed metabolic characterisation or without a lean control group and longer-term recording of the GH-IGF-1 axis after metabolic surgery [9, 15, 19–22]. The present study closely monitored the post-surgical time course of changes in the GH-IGF1 axis in comprehensively phenotyped individuals with class 3 obesity to elucidate factors associated with the reversal of altered GH-IGF-1 secretion. We hypothesised that post-surgical GH-IGF-1 axis improvements relate to the restoration of adipose tissue dysfunction and insulin sensitivity via changes in the secretion patterns of adipokines and pro-inflammatory cytokines.

## Methods

### Study population

We studied people with obesity of Caucasian origin (OBE, *n* = 79) before and 2, 12, 24 and 52 weeks after sleeve gastrectomy (*n* = 30) or gastric bypass surgery (*n* = 49). Healthy Caucasians without obesity were examined once as controls (CON, *n* = 24). T2D was present in 19 of the participants with obesity. All participants were non-smokers, engaged only in light physical activity and neither had previous pituitary disease (including known GH deficiency) or surgery nor received GH replacement. Data of some participants were part of previous reports of the BARIA_DDZ cohort [18, 23]. They provided informed written consent to this registered clinical cohort study (NCT01477957), which was approved by the ethics board of Heinrich-Heine University and University Hospital Düsseldorf and the ethics board of the North Rhine regional physicians’ association.

### Clamp test

Each participant underwent 3 h hyperinsulinemic-euglycemic clamps employing the isotopic dilution technique using D-[6,6-^2^H_2_]glucose for measuring whole-body (mainly skeletal muscle) insulin sensitivity from insulin-stimulated rate of glucose disposal (clamp-Rd) [18, 23]. Fasting hepatic insulin sensitivity (HIS) was calculated by the formula: 100/[fasting endogenous glucose production (EGP)*fasting insulin] [23]. Adipose tissue insulin resistance was assessed in the fasted state from Adipo-IR, calculated as FFA_fasting_*insulin_fasting_, [24, 25] and during the hyperinsulinemic-euglycemic clamp from the percent suppression of FFA concentrations, calculated as [FFA_fasting_ -FFA_clamp360 min_]*100/FFA_fasting_ [26, 27]. Steady-state rates of glucose appearance (Ra) were calculated as [tracer infusion rate]*[tracer enrichment]/[percent tracer enrichment in plasma]-[tracer infusion rate] [28]. While in the fasted state, EGP equals Ra, clamp-Ra and -Rd were calculated using Steele’s steady state equations.

### Blood analyses

Blood samples were collected before and during clamps for measuring hormones and metabolites. Metabolites, insulin, C-peptide, hsCRP, transforming growth factor β1 (TGFβ1), interleukin 1 receptor antagonist (IL-1ra), CC chemokine ligand 18 (CCL18), total adiponectin and leptin were quantified as described [29–31]. In vitro lipolysis was prevented by collecting blood into orlistat-containing vials [32] for microfluorimetrical FFA quantification (Wako Chem USA Inc. Osaka, Japan). Serum concentrations of GH, IGF-1, IGFBP1 and IGFBP3 were measured by ELISA (Quantikine® ELISA immunoassay, R&D Systems, Inc., MN, USA) in samples obtained in the morning after overnight fasting. The intraassay coefficients of variations (CVs) for GH, IGF-1, IGFBP1 and IGFBP3 were 3.6%, 2.3%, 2.7% and 1.4%, respectively, and interassay CVs for GH, IGF-1, IGFBP1 and IGFBP3 were 6.5%, 3.5%, 6.8% and 9.6%, respectively.

### Statistical evaluation

Normally distributed parameters are presented as means ± SD or means ± SEM, otherwise as median (Q1;Q3). Not-normally distributed data were log_e_-transformed to achieve near-normal distribution. Statistical analyses using covariance pattern model for repeated measures analysis were performed. Analysis of covariance (ANCOVA) models of the cohort of participants with obesity as well as regression models were adjusted for age and sex and performed using SAS (version 9.4; SAS Institute, Cary, NC, USA).

## Results

### OBE exhibit lower circulating GH, but not IGF-1 levels than CON

**Fig. 1:**
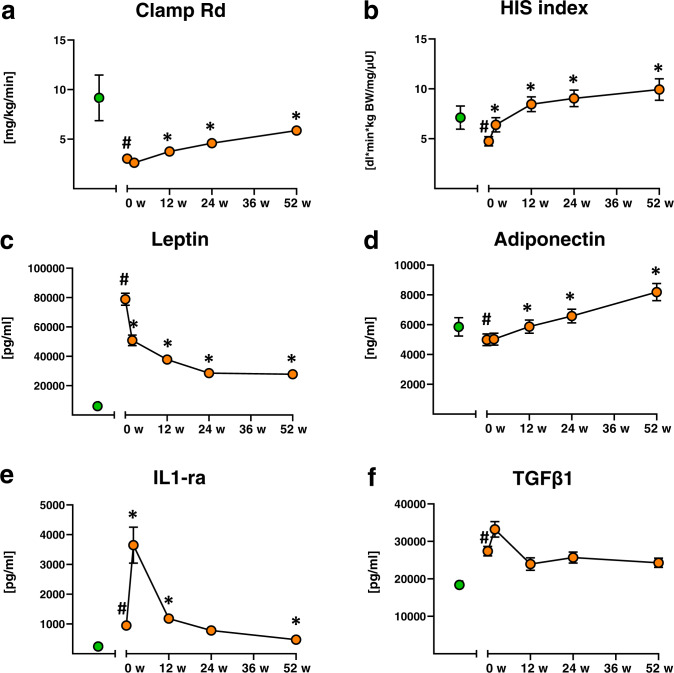
Time course of changes in insulin sensitivity, adipokines and cytokines. Changes in rate of glucose disposal during the hyperinsulinemic-euglycemic clamp (clamp-Rd) (**a**), hepatic insulin sensitivity index (HIS) (**b**), leptin (**c**), total adiponectin (**d**), interleukin 1 receptor antagonist (IL-1ra) (**e**) and transforming growth factor β1 (TGFβ1) (**f**). Control humans depicted by green circles, people with obesity before (0 w) and 2, 12, 24 and 52 weeks after surgery depicted by orange circles. Data are mean ± SEM, ^#^*p* < 0.05 vs controls, *p < 0.05 vs obese at baseline (0 w).

**Fig. 2:**
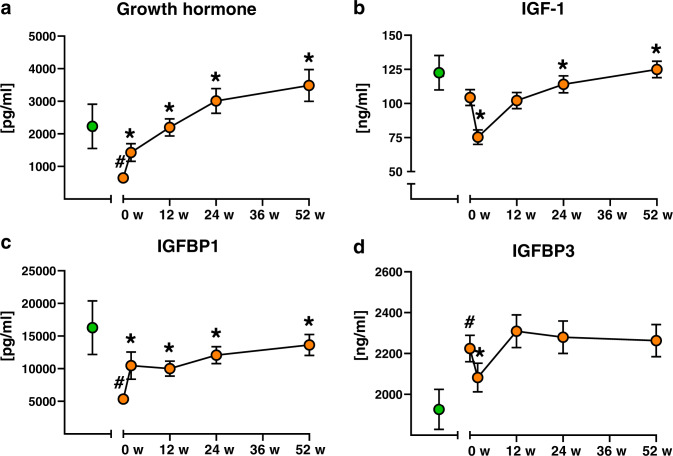
Time course of changes in growth hormone and its mediators. Changes in growth hormone (GH) (**a**), insulin-like growth factor-1 (IGF-1) (**b**), IGF-1 binding protein 1 (IGFBP1) (**c**) and IGFBP3 (**d**). Control humans depicted by green circles, people with obesity before (0 w) and 2, 12, 24 and 52 weeks after surgery depicted by orange circles. Data are mean ± SEM, ^#^*p* < 0.05 vs controls, *p < 0.05 vs obese at baseline (0 w).

Before surgery (baseline), OBE had similar age, but higher fasting glycemia and Adipo-IR as well as lower clamp-Rd and HIS when compared to CON (Table 1, Fig. 1a, b). They also had higher plasma FFA but comparable triglycerides (Table 1). Serum insulin, leptin and IGFBP3 were higher, IGF-1 similar, while GH and IGFBP1 were lower in OBE (Table 1, Fig. 2a–d). BMI was higher in individuals with T2D compared to those without T2D, but levels of leptin, GH, IGF-1, IGFBP1 and IGFBP3 were comparable (Suppl. Fig. 1).

**Table 1:** Participants’ characteristics.

Parameter	CON	OBE				
		Baseline	2 w	12 w	24 w	52 w
*N* (male)	24 (10)	79 (16)	66 (14)	76 (15)	73 (16)	68 (15)
Age (years)	43.7 ± 11.8	40.3 ± 9.2				
BMI (kg/m^2^)	24.3 ± 3.1	50.8 ± 6.3^a^	47.0 ± 5.9^b^	41.7 ± 5.8^b^	37.7 ± 5.7^b^	33.8 ± 5.5^b^
Glucose (mg/dl)	84 ± 8	98 ± 26^a^	92 ± 23^b^	85 ± 16^b^	82 ± 12^b^	80 ± 10^b^
Insulin (μU/ml)	6(3;8)	21(17;31)^a^	19(14;23)^b^	12(8;17)^b^	9(7;13)^b^	8(5;11)^b^
HbA1c (%)	5.2 ± 0.4	5.9 ± 0.9^a^	5.5 ± 0.8^b^	5.3 ± 0.5^b^	5.2 ± 0.5^b^	5.1 ± 0.4^b^
FFA (µmol/l)	388 (316; 630)	679 (509; 822)^a^	1003 (864; 1169)^b^	658 (566; 833)	592 (460; 779)	466 (357; 618)^b^
Triglycerides (mg/dl)	105 ± 91	131 ± 63	129 ± 46	115 ± 35	101 ± 31^b^	88 ± 30^b^
hsCRP (mg/dl)	0.2 ± 0.1	1.0 ± 0.8^a^	1.1 ± 1.8	0.6 ± 0.4^b^	0.4 ± 0.4^b^	0.3 ± 0.5^b^
CCL18 (pg/ml)	37682 ± 14453	74662 ± 25808^a^	74502 ± 23446	73024 ± 26199	67003 ± 22096^b^	49535 ± 16623^b^
Adipo-IR (AU)	2538 ± 1651	17064 ± 10720^a^	21764 ± 14247^b^	8876 ± 5492^b^	7732 ± 10000^b^	4443 ± 37097^b^
FFA suppression (%)	90.3 ± 5.3	85.4 ± 12.7	59.3 ± 21.7^b^	90.5 ± 7.2^b^	94.3 ± 3.5^b^	94.6 ± 3.5^b^

Mean ± SD or median (Q1;Q3).

*Adipo-IR* adipose tissue insulin resistance index, *BMI* body mass index, *CCL18* CC chemokine ligand 18, *FFA suppression* (FFAfasting-FFAclamp 360 min)*100/FFAfasting, *CON* lean healthy controls, *FFA* plasma free fatty acids, *hsCRP* high-sensitive C-reactive protein, *HIS* hepatic insulin sensitivity index (100/(fasting endogenous glucose production*fasting insulin)), *OBE* people with obesity at baseline.

^a^*p* < 0.05 vs CON.

^b^*p* < 0.05 vs OBE at baseline.

### GH rapidly and continuously rises, while IGF-1 levels transiently decrease upon metabolic surgery

At 2 weeks after surgery, body weight loss of 10 ± 3 kg was paralleled by a transient increase in FFA, Adipo-IR and HIS, but no change in whole-body insulin sensitivity (Table 1, Fig. 1a, b). Serum insulin, insulin-mediated percent FFA suppression and leptin decreased in OBE early after surgery (Table 1, Fig. 1c). In parallel, the pro-inflammatory biomarkers, IL-1ra and TGFβ1, were transiently higher (*p* < 0.01) or tended to be higher (*p* = 0.11), respectively, compared to baseline (Fig. 1e, f).

Until 52 weeks after surgery, OBE exhibited an average weight loss of 33% and continuous improvements in whole-body, adipose tissue and HIS (Fig. 1a, b, Table 1). Similarly, glycemia, Adipo-IR, FFA, hsCRP and IL-1ra were normalised at 52 weeks (Table 1, Fig. 1e). Serum insulin, CCL18 and leptin levels were decreased by 63%, 34% and 65% at 52 weeks, respectively (Table 1, Fig. 1c), while total adiponectin markedly increased (Fig. 1d). Time courses of changes in BMI, insulin, glucose, HbA1c, triglycerides, FFA, hsCRP, Adipo-IR and IL-6 have been reported in a previous analysis of the BARIA_DDZ cohort [18].

At 2 weeks, serum GH and IGFBP1 were already increased, but IGF-1 and IGFBP3 levels dropped by 28% and 6% from baseline, respectively (Fig. 2b, d). During the follow-up, GH and IGFBP1 levels continuously rose and were higher or equal to that of CON, respectively, at 52 weeks (Fig. 2a, c). IGF-1 rose only later, at 24 and 52 weeks after surgery (Fig. 2b).

In participants with T2D, body weight loss was lower, but improvement of adipose tissue insulin sensitivity was higher at 24 and 52 weeks after surgery compared to participants without T2D (Suppl. Fig. 1). Increases in IGFBP1 at 12 weeks and IGFBP3 at 52 weeks were higher in individuals without T2D compared to participants with T2D (Suppl. Fig. 1). Despite lower peripheral insulin sensitivity at baseline (*p* < 0.0001, data not shown), participants with T2D had greater improvements in insulin-stimulated Rd at 2, 12, and 52 weeks compared to participants without T2D (*p* = 0.007, *p* = 0.0006 and *p* = 0.02, respectively, data not shown).

Of note, there were no differences in the time courses of changes of BMI, hepatic and adipose tissue insulin sensitivity as well as GH, IGF-1, IGFBP1, IGFBP3 and leptin concentrations between participants undergoing sleeve gastrectomy and gastric bypass surgery (Suppl. Fig. 2).

### Reversal of the GH/IGF1 system relates to improved FFA and adipose tissue insulin sensitivity, but not whole-body insulin sensitivity

Multiple regression analysis adjusted for age and sex revealed no association between the long-term improvement in GH concentrations and changes in body weight, peripheral or HIS, glycemia or leptin levels, but a negative association with the reduction in insulinemia, FFA, and Adipo-IR (Table 2). The 52-week increase in IGF-1 related positively to the changes in leptin and insulin and negatively to the changes in hsCRP (Table 2). Of note, the transient lowering of IGF-1 at 2 weeks related to the increase in hsCRP, while the decrease in IGFBP3 related to the increase in Adipo-IR and hsCRP (*p* = 0.04, *p* = 0.04 and *p* = 0.03, respectively, data not shown). The 2-week increase of IGFBP1 related positively to the higher FFA concentrations (*p* = 0.008, data not shown).

**Table 2:** Multiple regression analysis (adjusted for age and sex) for changes in serum GH and IGF-1 levels over 52 weeks after surgery.

Dependent	Parameter	Estimate	*P* value
Change in lnGH from baseline to 52 weeks (Δ lnGH)	Δ body weight	−0.017	0.395
	Δ fasting blood glucose	−0.015	0.384
	Δ HbA1c	−0.585	0.219
	Δ lnFFA	−0.941	**0.027**
	Δ lnInsulin	−1.410	**0.027**
	Δ lnClamp-Rd	1.725	0.117
	Δ lnLeptin	−0.098	0.757
	Δ lnhsCRP	−0.325	0.066
	Δ lnAdipo-IR	−1.479	**0.002**
	Δ FFAsuppr	0.111	**0.012**
Change in lnIGF-1 from baseline to 52 weeks (Δ lnIGF-1)	Δ body weight	0.002	0.666
	Δ fasting blood glucose	−0.002	0.428
	Δ HbA1c	0.011	0.912
	Δ lnFFA	0.144	0.108
	Δ lnInsulin	0.243	**0.042**
	Δ lnClamp-Rd	−0.044	0.832
	Δ lnLeptin	0.209	**0.007**
	Δ lnhsCRP	−0.093	**0.018**
	Δ lnAdipo-IR	0.204	0.091
	Δ FFAsuppr	0.007	0.471

*FFA* free fatty acids, *GH* growth hormone, *IGF-1* insulin-like growth factor 1, *hsCRP* high-sensitive C-reactive protein, Δ refer to the difference between values at baseline and 52 weeks, *ln* natural logarithm, *clamp-Rd* insulin-stimulated rate of glucose disposal.

Bold values identify statistical significance (*P* < 0.05).

## Discussion

This study demonstrates that the reversal of alterations of the GH-IGF-1 axis after bariatric surgery relates to improvements in adipose tissue function, but not whole-body and HIS. In particular, normalisation of the obesity-related so-called “functional hyposomatotropism” by surgical weight loss is associated with the reduction in lipid availability, adipose tissue insulin resistance and low-grade inflammation, underlining an important role of adipose tissue also for the regulation of GH-IGF-1 axis in metabolic diseases.

First, this study found lower GH concentrations in humans with obesity than in lean humans in the setting of similar IGF-1 concentrations, indicating a preserved IGF-1 feedback mechanism. Alterations of the GH-IGF-1 axis in obesity have been demonstrated previously by reduced fasting or stimulated GH [8, 33] and controversial data has been reported for IGF-1 concentrations [3, 34]. The latter is likely due to the complex regulation of IGF-1, which in addition to GH involves several other factors, e. g. hypothalamic neuropeptides, ghrelin, insulin, FFA, macronutrients and IGFBPs [14, 35].

The observed increase in GH and IGF-1 levels confirms data at 6- and 12-m follow-up from other prospective bariatric surgery cohorts [9, 19, 22, 36], while the novel data for the 2-w timepoint allows further insights into short-term changes. These revealed a transient reduction in IGF-1 and IGFBP3 levels, which associated with increased hsCRP, suggesting a tight link between inflammatory processes and IGF-1 signaling. This is supported by data showing that interleukin 1 (IL-1), tumour necrosis factor α and the mitogen activated protein kinase pathway regulate IGF-1 and IGFBPs in cross-sectional human [37] and mechanistic rodent studies [38]. The present study also showed a negative association between circulating GH and insulin levels in line with the observation of fasting insulin as a predictor of integrated 24-h GH release [39]. Indeed, insulin infusion lowers the GH response to GH-releasing hormone via the pituitary [40], suggesting an IGF-1-like effect [41]. In addition, the postoperative GH response in persons with obesity undergoing bariatric surgery seems to be mainly modulated by insulin [21]. GH is also a determinant of lean (muscle) mass after metabolic surgery [42] and exercise training [43]. In this context, the improved insulin and GH levels could be responsible for the increase in muscle lipid oxidation pathways and epigenetic regulation of muscle differentiation, as assessed from Gene Ontology analyses in people with obesity at 52 weeks after bariatric surgery [18]. Despite lack of lean body mass measurements in the present study, the improved muscle differentiation and GH levels after bariatric surgery possibly contribute to preservation of lean mass after surgery as seen with GH treatment during hypocaloric diet [44].

Of note, this study uncovers a direct link between the surgically-induced improvement in GH levels and adipose tissue insulin sensitivity as well as lipid availability. Adipose tissue dysfunction has developed as the key mechanism underlying the pathophysiology of whole-body insulin resistance mediated by lipotoxicity and low-grade inflammation [27, 45, 46]. GH action also targets lipolysis, lipogenesis as well as adipocyte proliferation, differentiation and function, including adipose tissue inflammation and adipokine secretion [47]. A possible mechanism underlying this link may be upregulation of the GH-dependent signal transducer and activator of transcription-5 phosphorylation as shown in skeletal muscle during acute FFA-suppression by acipimox [48], which is mediated by adipocyte JAK2 signaling [49]. Notably, presence of T2D does not seem to play a relevant role for the reversal of the alterations of the GH-IGF-1 axis, as most observed changes were not dependent on T2D status and no association was found between glycemia and GH-IGF-1 changes. This suggests that early metabolic alterations in insulin sensitivity and adipose tissue function rather than overt diabetes and hyperglycemia are linked to changes in the GH system [50].

Furthermore, the adipokine leptin, which signals adipose tissue mass and energy balance to the brain, also inhibits GH secretion [15, 51] and contributes to IGF-1 regulation [16] and could therefore account for the changes in the GH-IGF axis induced by surgical weight loss. While this study confirms the substantial and continuous improvement of hyperleptinemia following metabolic surgery, no relationship was found between the decrease in circulating leptin and the improvement of GH levels. Of note, leptin secretion may be inhibited under conditions of greater insulin sensitivity [52, 53], so that the present results suggest a dissociation between adipose tissue and skeletal muscle insulin sensitivity with regard to leptin control of the GH-IGF-1 axis. Of note, leptin levels remained elevated at 52 weeks after surgery when compared to lean healthy controls, while the circulating GH concentrations at 52 weeks reached those of the nonobese control group. In line with previous reports, the type of metabolic surgery did neither affect change in GH and leptin [22] nor in body weight loss as well as in improvements of hepatic and adipose tissue insulin sensitivity [18].

Finally, IGFBP1 is negatively associated with impaired glucose tolerance [54] and obesity [55] and serves as a marker of HIS [56], while IGFBP3 correlates directly with hepatic insulin resistance and diabetes incidence [57, 58]. Thus, the long-term changes in IGFBP1 and IGFBP3 after metabolic surgery, as seen in the present study, reflect the glucometabolic improvement in line with previous reports [20]. The transient changes in IGFBP1 and IGFBP3 at 2 weeks after surgery and their relationship to changes in FFA and hsCRP point to a previously unknown link between IGFBPs and adipose tissue function and low-grade inflammation in obesity. This may be due to IGF-1-independent effects of IGFBP3 and IGFBP1 on adipose tissue, such as action via type V TGFβ receptors [59, 60].

In conclusion, the present findings provide detailed insights into dynamic endocrine changes in persons with obesity following metabolic surgery by linking reversal of the dysregulation of the GH-IGF-1 axis to adipose tissue metabolism and function. Specifically, these results point to a future role of modulating GH and its mediators in the treatment of obesity and obesity-related disorders.

## Supplementary information

Supplement

## Data Availability

The datasets used and analysed during the current study are available from the corresponding author on reasonable request.
