# Supplementary material for: Metabolic surgery-induced changes of the growth hormone system relate to improved adipose tissue function
Source: Int J Obes (Lond). 2023 Mar 23;47(6):505–11. doi: 10.1038/s41366-023-01292-7 (PMC10212759; doi:10.1038/s41366-023-01292-7)

**Supplementary material**

**Suppl. Fig. 1.** **Time course of changes in body mass index, adipose tissue and hepatic insulin sensitivity as well as serum leptin, growth hormone, IGF-1, IGFBP1 and IGFBP3 in participants with obesity with and without type 2 diabetes after bariatric surgery.** Participants without type 2 diabetes depicted with white bars, participants with type 2 diabetes depicted with dark grey bars. Data are mean ± SEM, #p<0.05 Δ change from baseline in nonT2D vs T2D, *p<0.05 nonTD2 vs T2D. FFAsuppr – insulin-mediated clamp free fatty acid suppression as (FFA_fasting_-FFA_clamp360 min_)*100/FFA_fasting_, HIS index – hepatic insulin sensitivity index, IGF-1- insulin like growth factor 1, IGFBP – insulin-like growth factor binding protein, nonT2D – participants with obesity without type 2 diabetes, T2D – participants with obesity and type 2 diabetes, w – weeks.

**
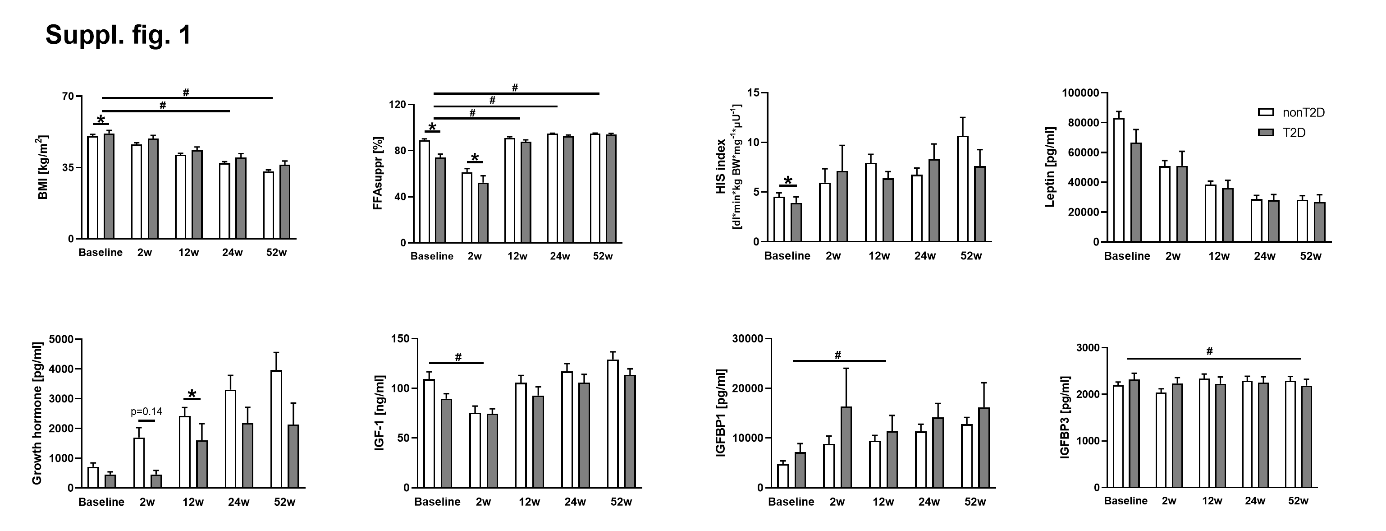
**

**Suppl. Fig. 2. Time course of changes in body mass index, adipose tissue and hepatic insulin sensitivity as well as serum leptin, growth hormone, IGF-1, IGFBP1 and IGFBP3 in participants with obesity undergoing sleeve gastrectomy and gastric bypass surgery.** Participants undergoing sleeve gastrectomy depicted by black bars, participants undergoing gastric bypass surgery depicted by light grey bars. Data are mean ± SEM, *p<0.05 in sleeve gastrectomy vs gastric bypass. FFAsuppr – insulin-mediated clamp free fatty acid suppression as (FFA_fasting_-FFA_clamp360 min_)*100/FFA_fasting_, HIS index – hepatic insulin sensitivity index, IGF-1- insulin like growth factor 1, IGFBP – insulin-like growth factor binding protein, w - weeks.


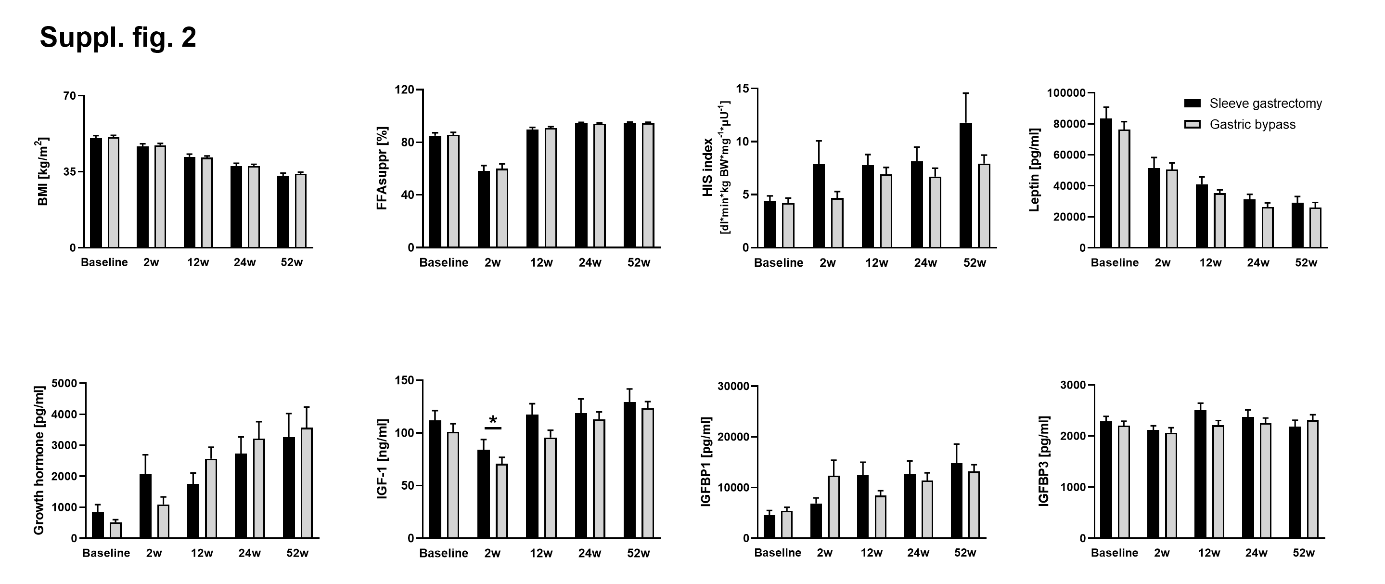

Supplement: Supplementary file 1 — Supplement [file 41366_2023_1292_MOESM1_ESM.docx]
